# Supplementary material for: Development of Chitosan Particles Loaded with siRNA for Cystatin C to Control Intracellular Drug-Resistant Mycobacterium tuberculosis
Source: Antibiotics (Basel). 2023 Apr 8;12(4):729. doi: 10.3390/antibiotics12040729 (PMC10135320; doi:10.3390/antibiotics12040729)
Supplement: Supplementary file 1 [file antibiotics-12-00729-s001.zip › antibiotics-2317496-supplementary.pdf]

Supplementary Material

Development of chitosan particles loaded with siRNA for cystatin C to control intracellular drug-resistant *Mycobacterium tuberculosis*

David Pires [1,2,†], Manoj Mandal [1,†], Ana I. Matos [3], Carina Peres [3], Maria João Catalão [1], José Miguel Azevedo-Pereira [1], Ronit Satchi-Fainaro, Helena F. Florindo [3] and Elsa Anes [1,*]

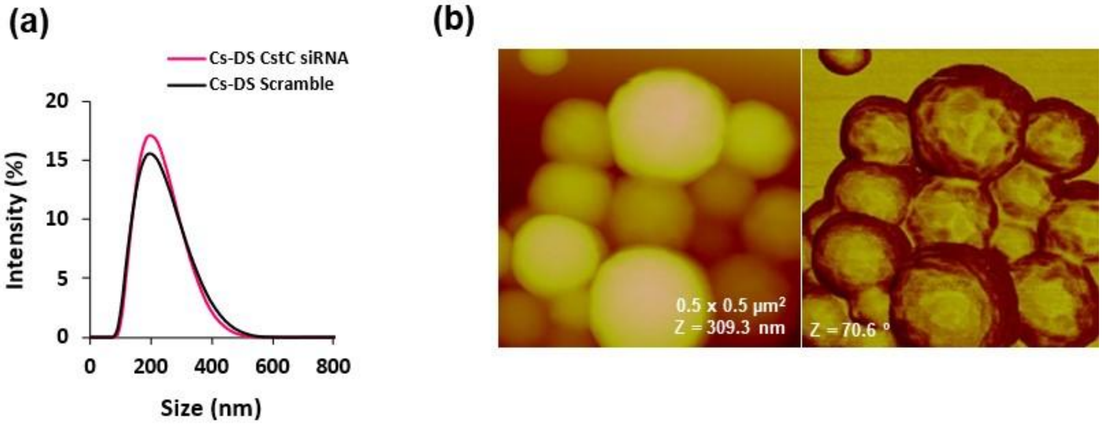

Figure S1. Physicochemical and morphological properties of Cs-DS. Dynamic light scattering analysis (a) and atomic force microscopy images (topography – left; phase – right) (b) showed a uniform-size polydispersity for slightly rough spherical particles.
